# Recently introduced biomarkers for screening of hepatocellular carcinoma: a systematic review and meta-analysis

**DOI:** 10.1007/s12072-012-9374-3

**Published:** 2012-05-12

**Authors:** Caroline D. M. Witjes, Susanna M. van Aalten, Ewout W. Steyerberg, Gerard J. J. M. Borsboom, Robert A. de Man, Cornelis Verhoef, Jan N. M. IJzermans

**Affiliations:** 5 Department of Hepatobiliary and Transplantation Surgery, Erasmus University Medical Centre, ’s Gravendijkwal 230, 3015 CE Rotterdam, The Netherlands; 1 Department of Hepatobiliary and Transplantation Surgery, Erasmus University Medical Centre, Rotterdam, The Netherlands; 2 Department of Public Health, Erasmus University Medical Centre, Rotterdam, The Netherlands; 3 Department of Hepato-Gastroenterology, Erasmus University Medical Centre, Rotterdam, The Netherlands; 4 Department of Surgical Oncology, Erasmus University Medical Centre, Rotterdam, The Netherlands

**Keywords:** Hepatocellular carcinoma, Biomarkers, Screening

## Abstract

**Purpose:**

Early detection of hepatocellular carcinoma (HCC) is essential for improved prognosis and long-term survival. To date, screening for HCC depends on serological testing (alpha-fetoprotein, AFP) and imaging (ultrasonography), both of which are not highly sensitive. A meta-analysis was performed to discuss recent developments in biomarkers that may be effective in screening for HCC.

**Methods:**

A systematic search of PubMed, Embase, and Web of Science was performed for articles published between January 2005 and October 2010, and focusing on biomarkers for HCC in urine, serum, or saliva. Data on sensitivity and specificity of tests were extracted from each included article and displayed with a summary ROC. A meta-analysis was carried out in which the area under the curve for each biomarker was used to compare the accuracy of different tests.

**Results:**

In seven well-defined studies, three biomarkers were identified for potential use, namely, Golgi protein 73 (GP73), interleukin-6 (IL-6), and squamous cell carcinoma antigen (SCCA). Comparison with AFP showed that GP73 was superior (*p* = 0.006; 95 % CL −0.23, −0.12), IL-6 was similar (*p* = 0.66; 95 % CL −0.31, 0.25), and SCCA was inferior to AFP (*p* = 0.001; 95 % CL 0.12, 0.23).

**Conclusion:**

GP73 is a valuable serum marker that seems to be superior to AFP and can be useful in the diagnosis and screening of HCC. Although GP73 may improve the detection and treatment of one of the most common malignancies worldwide, additional research is required.

## Introduction

The incidence of hepatocellular carcinoma (HCC) is rising in many countries [1]. Alpha-fetoprotein (AFP) levels and ultrasonography are widely applied for HCC screening. However, AFP alone has a sensitivity of 60 % at a cut-off value of 20 ng/mL, and ultrasonography has a sensitivity of 65–80 % with a specificity ≥90 % when used as a screening test [1].

The lack of efficacious tests necessitates investigation for new HCC markers. Recent studies have focused on tests that can detect HCC, including tests for DCP, also known as prothrombin induced by vitamin K absence II (PIVKA II), the ratio of glycosylated AFP (L3 fraction) to total AFP, alpha fucosidase, glypican 3, and HSP-70. However, as sensitivity and specificity values of these serological markers were low, they proved to be inadequate for HCC screening purposes, even when combined [1].

General criteria for effective disease screening have been proposed by the World Health Organization (WHO). These criteria are as follows: the disease screened for should represent a major cause of death, the natural history of the disease should be well characterized, screening for the disease should be cost-effective, and the screening test should be acceptable to the population. In addition, facilities for diagnosis and treatment should be available, and there should be a treatment for the disease that improves the outcome if the disease is detected at an early stage [2]. These WHO criteria are met for HCC [1, 3].

To define the present state-of-the-art technology for HCC screening, we initiated a systematic review and meta-analysis, and discuss biomarkers most likely to be introduced as new instruments for HCC screening.

## Materials and methods

### Literature search strategy

A systematic search of PubMed, Embase, and Web of Science was performed for articles published between January 2005 and October 2010 (cut-off date 1 October 2010) and relevant to HCC biomarkers in urine, serum, or saliva. In 2005, Bruix et al. [1, 4] published the AASLD guidelines, which they updated in 2011. However, with respect to screening tests, their paper did not report on new findings [1]. Therefore, in the present study a new literature search was initiated based on the search terms listed in Table 1.

**Table 1:** Terms used in the systematic search for the present review

Database	Search terms
*Pubmed*	(hepatocellular carcinoma[mesh] OR hepatoma*[tw] OR liver cell neoplasm*[tw] OR hepatocellular neoplasm*[tw] OR liver cell cancer*[tw]
	OR hepatocellular cancer*[tw] OR liver cell tumo*[tw] OR hepatocellular tumo*[tw] OR liver cell carcinom*[tw] OR hepatocellular carcinom*[tw]) AND
	(biological markers[mesh] OR Biomarker*[tw] OR Biological Marker*[tw] OR Biologic Marker*[tw] OR Biochemical Marker*[tw] OR Immunologic Marker*[tw]
	OR Immune Marker*[tw] OR Laboratory Marker*[tw] OR Serum Marker*[tw] OR Clinical Marker*[tw]) AND (blood[mesh] OR blood[sh] OR blood[tw] OR
	serum*[tw] OR plasm[tw] OR plasma[tw] OR urine[mesh] OR urine[sh] OR urine*[tw] OR saliva[tw]) NOT (animals[mesh] NOT humans[mesh]) AND
Limits	Publication date: 2005-3000 OR entrance date: 2005-3000 AND
	Language: English OR Dutch
*Embase*	(((‘liver cell’ OR hepatocell*) NEAR/3 (neoplasm* OR cancer* OR tumo* OR carcinom*)):ti,ab,de) AND (marker/exp OR (biological NEAR/3 marker*):ti,ab,de
	OR biomarker*:ti,ab,de) AND (blood/exp OR blood:ti,ab,de OR serum*:ti,ab,de OR plasm:ti,ab,de OR plasma:ti,ab,de OR urine*:ti,ab,de OR saliva:ti,ab,de)
Limits	Publication date: 2005-present AND
	Human
	Language: English OR Dutch
*Web of Science*	(hepatoma* OR liver cell neoplasm* OR hepatocellular neoplasm* OR liver cell cancer* OR hepatocellular cancer* OR liver cell tumo* OR hepatocellular
	tumo* OR liver cell carcinom* OR hepatocellular carcinom*) AND (Biomarker* OR Biological Marker* OR Biologic Marker* OR Biochemical Marker* OR
	Immunologic Marker* OR Immune Marker* OR Laboratory Marker* OR Serum Marker* OR Clinical Marker*) AND (blood OR serum* OR plasm OR plasma
	OR urine* OR saliva) NOT (animal* NOT human*)
Limits	Publication date: 2005-present AND
	Language: English

### Literature screening

**Fig. 1:**
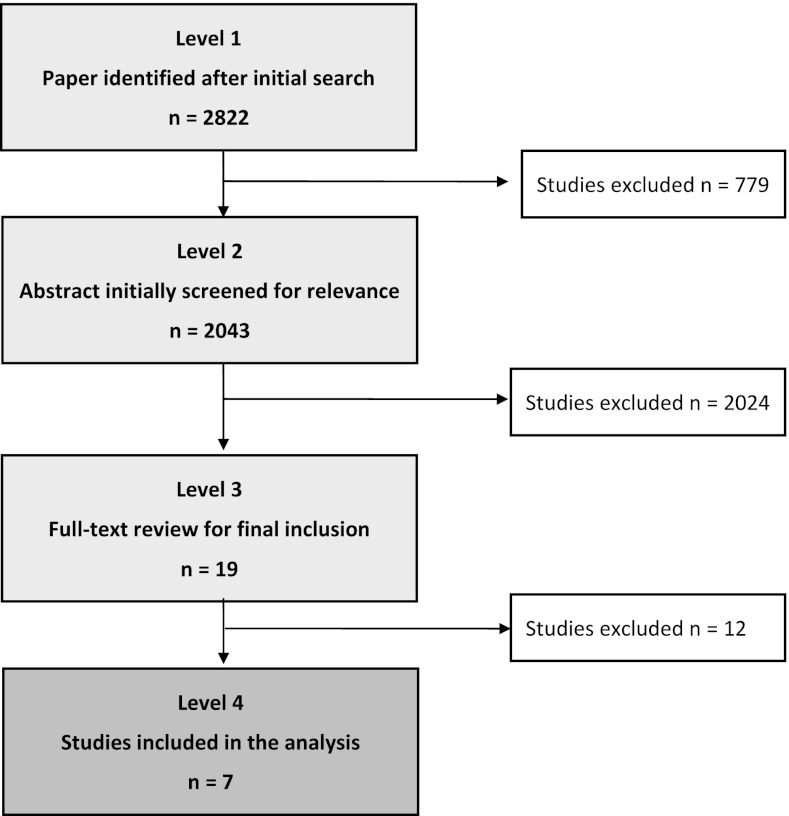
Flow diagram showing selection of the seven articles

Studies were evaluated for their relevance to our present topic. Study selection was accomplished through four levels of study screening (C.D.M.W. in consensus with S.M.A) (Fig. 1). At level 1, studies were excluded for the following reasons: review, letters, case reports, editorials, and comments. At level 2, abstracts of all the studies accepted at level 1 were reviewed for relevance. The full text was obtained for relevant papers, as well as any citations for which a decision could not be made from the abstract. At level 3, inclusion required a control group with ≥10 cirrhotic patients (hepatitis B and/or hepatitis C and/or alcohol abusers), ≥10 patients with a HCC, and ≥10 confirmed healthy persons. Finally, at level 4, those studies that tested the biomarker in a second independent population were included together with the studies that were a continuation of studies included at level 3. All studies without repeated measurements, as validation of their method, were excluded.

### Data extraction and critical appraisal

From each included article, we extracted data on study design, study population, and test results. The level of evidence of each article was scored using the Oxford Centre for Evidence-based Medicine Level of Evidence scale [5].

Data on sensitivity and specificity were extracted from each included article. If percentages were not reported, the sensitivity and specificity at several cut-off points were taken from the ROC curve in the included manuscripts.

### Statistics

Sensitivities and specificities of the included studies were logistically transformed, and a linear regression line was fitted through the resulting points. This line was then back-transformed to obtain the summary ROC (sROC) curve, according to the method described by Littenberg and Moses in 1993 [6]. A conventional ROC curve describes the impact of threshold in a single patient population. The sROC curve, a compact description of the accuracy of the diagnostic test, describes the test in many populations. Note that we did not extrapolate the curve past the range of empiric data.

The area under the curve (AUC) for the biomarkers of the included studies was taken from the reports. For each biomarker, the pooled AUC was calculated using the inverse standard errors as weights. This pooled AUC, together with their similarly pooled standard error, was used to compare the accuracy of the diagnostic tests. AFP was considered as a reference for comparison to the other markers and was compared with the pooled AUC of each new biomarker using Student’s *t* test. SAS software (SAS system 9.2, SAS Institute, Cary, NC, USA) was used to perform the statistical analyses. A result was considered statistically significant at a *p* value of <0.05.

## Results

Among 2,822 articles identified by the initial search, seven were within the scope of the study (Fig. 1) [7–13]. Two articles described Golgi protein 73 (GP73) as a HCC biomarker [7, 8], two described interleukin-6 (IL-6) [9, 10], and three described squamous cell carcinoma antigen (SCCA) [11–13]. All identified studies provided level 2b evidence on the Oxford Level of Evidence scale and included a control group with ≥10 cirrhotic patients (hepatitis B and/or hepatitis C and/or alcohol abusers), ≥10 patients with a HCC, and ≥10 confirmed healthy persons [5].

### GP73

GP73, also named Golgi phosphoprotein 2 (GOLPH2), is a 400-amino acid, 73 kDa transmembrane glycoprotein that normally resides within the *cis*-Golgi complex [7].

Marrero et al. tested GP73 in the sera of 352 patients, of whom 144 had HCC, 152 had cirrhosis, and 56 did not have any disease [7]. At the optimal cut-off point of 10 relative units (RU), the sensitivity of GP73 was 62 %, with a specificity of 88 %.

**Fig. 2:**
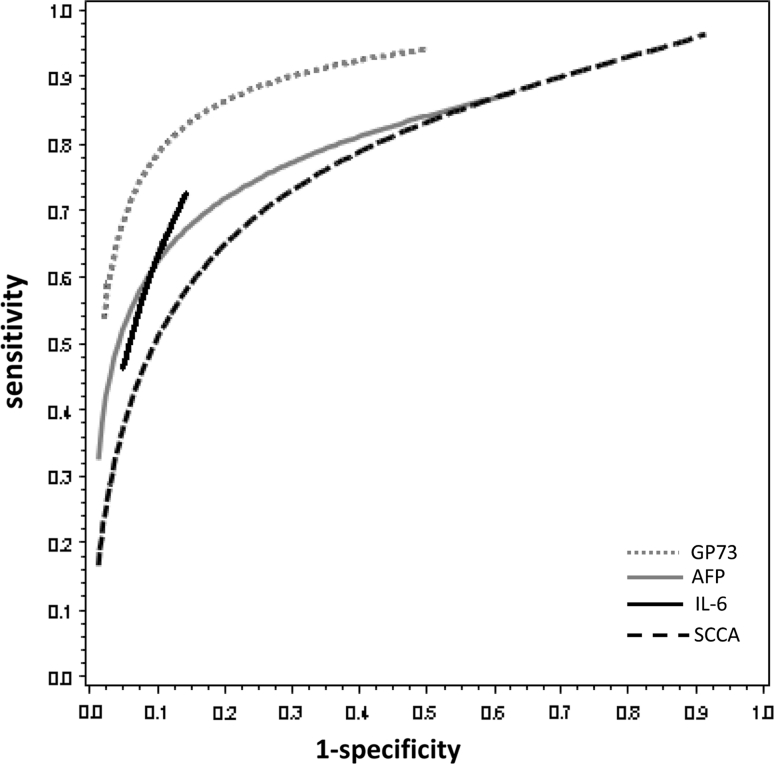
The sROC with the sensitivity and 1-specificity of GP73, AFP, IL-6, and SCCA

A recent study by Mao et al. tested GP73 in the sera of 4,217 subjects: 789 with HCC, 427 who were HBV or HCV carriers, 614 with cirrhosis, and 1,690 healthy controls [8]. GP73 sensitivity was 74.6 % and specificity was 97.4 % at an optimal cut-off value of 8.5 RU. The sROC of GP73 in these studies is shown as the gray dotted line in Fig. 2.

### IL-6

IL-6 is a pleiotropic cytokine playing a central role in hematopoiesis, and in the differentiation and growth of a number of cells with different histological origins [9, 10]. The expression of IL-6 on hepatocytes, its upregulation by hepatitis B virus X protein, and its increased hepatic expression in liver cirrhosis have made IL-6 an intriguing cytokine to study in HCC [9].

Porta et al. [9] studied IL-6 in the sera of 90 patients: 30 with HCC, 30 with cirrhosis, and 30 healthy subjects. At the cut-off of 12 pg/mL, they found a sensitivity of 73 % with a specificity of 87 %.

Hsia et al. [10] also studied IL-6 in the sera of 128 patients, of whom 26 patients had HCC, 50 had chronic HBV or HCV infection, and 29 were without any disease (healthy controls). The authors found a sensitivity of 46 % with a specificity of 95 % for IL-6 at a cut-off of 3 pg/mL. The sROC of IL-6 of these studies is shown as the black straight line in Fig. 2.

### SCCA

SCCA, a component of the high molecular weight serine protease inhibitors named serpins, is physiologically expressed in the squamous epithelia [11–13]. Increased levels have been detected in several epithelial cancers such as those of the head, neck, cervix, and lung [11–13].

Giannelli et al. [11] tested SCCA in the sera of 251 patients: 120 with HCC, 90 with cirrhosis, and 41 healthy subjects. At an SCCA cut-off of 0.368 ng/mL, the sensitivity was 84 % with a specificity of 48 %.

In 2007, Giannelli et al. [12] reported on serum SCCA testing in 961 patients at a cut-off of 3.8 ng/mL; a sensitivity of 42 % with a specificity of 83 % was found.

In 2008, Hussein et al. [13] evaluated SCCA in the sera of 94 patients, including 49 patients with HCC, 30 with chronic liver disease without HCC, and 15 healthy persons. They used several cut-off points for SCCA: 100 % sensitivity and 7 % specificity were found at cut-off 0.3 ng/mL; 78 % sensitivity and 84 % specificity, at cut-off 1.5 ng/mL; and 39 % sensitivity and 100 % specificity were found at cut-off 3.5 ng/mL. The sROC of SCCA in these three studies is shown as the black dotted line in Fig. 2.

### AFP

Under physiological conditions, AFP is a fetal-specific glycoprotein with a molecular weight of around 70 kDa. It is synthesized primarily by cells of the embryonic liver, of the vitelline sac, and of the fetal intestinal tract in the first trimester of pregnancy [14]. The serum concentration of AFP declines rapidly after birth, and its expression is repressed in adults [14]. In the pathological state of chronic liver disease, particularly, that associated with a high degree of hepatocyte regeneration, AFP can be expressed in the absence of cancer [14]. All studies compared the performance characteristics of their biomarker to those of AFP in differentiating HCC from non-malignant chronic liver disease [14]. The seven articles defined within the scope of this study all tested serum AFP in their population. The sensitivity and specificity for AFP are summarized in Table 2. The sROC of AFP of the seven studies is shown as the gray straight line in Fig. 2.

**Table 2:** Characteristics and outcome measures of the included studies describing serum AFP levels in the patients tested

Authors	Year published	No. of patients	Cut-off value AFP (ng/mL)	Sensitivity (%)	Specificity (%)
Marrero et al. [7]	2005	352	112	25	97
Mao et al. [8]	2010	4,217	35	58	85
Porta et al. [9]	2008	90	12.8	63	88
Hsia et al. [10]	2007	128	20	62	88
Giannelli et al. [11]	2005	251	12.6	45	87
Giannelli et al. [12]	2007	961	18.8	41	94
Hussein et al. [13]	2008	94	7.7	90	93

*AFP* alpha-fetoprotein

### SROC

The sROC is a method for summarizing discrepant data on the accuracy of diagnostic technologies; it summarizes the central tendency of a set of accuracy reports.

Comparing the ‘gold standard’ with the three new biomarkers displayed that GP73 was superior to AFP (*p* = 0.006; 95 % CL −0.23, −0.12), IL-6 was similar to AFP (*p* = 0.66; 95 % CL −0.31, 0.25), and SCCA was inferior to AFP (*p* = 0.001; 95 % CL 0.12, 0.23).

## Discussion

This systematic review has attempted to correlate recently discovered biomarkers for HCC screening with AFP. Our findings suggest an advantage of GP73 over AFP as a serum marker for HCC screening. In our review process, many papers were excluded because of limitations in study design: mostly because of a poor definition of the underlying etiology and the absence of a healthy control group. Due to our rigorous selection criteria, the review was limited to seven studies with level 2b evidence, all testing their biomarker in serum [5]. Preferably, HCC screening should be performed using a non-invasive diagnostic test. Although the field of tumor markers in HCC is rapidly evolving, no ideal marker tested with a proper study design currently exists.

Since HCC is among the cancers with the worst prognosis, early diagnosis and treatment are essential for effective treatment [1]. The use of serological markers in patients at highest risk for developing HCC may decrease HCC mortality. However, for many years AFP has been the only standard serum marker for the detection of HCC, despite its unsatisfactory sensitivity [1]. Therefore, several new biomarkers (such as GP73, IL-6, and SCCA) have been investigated for their diagnostic accuracy and potential clinical application.

Giannelli et al. reported SCCA to be a good biomarker for discriminating early HCC from liver cirrhosis. Combining the three studies reporting on SCCA in a meta-analysis, we found SCCA to be inferior to AFP (*p* = 0.001).

In this systematic review, our meta-analysis of publications reporting on IL-6 showed that the accuracy of IL-6 was similar to that of AFP (*p* = 0.66).

Recent studies have identified serum GP73 as a potential biomarker for HCC. The study of Marrero et al. showed GP73 to be promising but had a small sample size [7]. A second study performed in medical centers in China and the USA showed GP73 to be a valuable tumor marker for HCC [8]. Combining both studies in our meta-analysis showed GP73 to be superior to AFP (*p* = 0.006). Although GP73 appears to be a better marker than AFP for diagnosing HCC, additional research is required that focuses on GP73.

Mao et al. found the elevation of serum GP73 to be modest in virus carriers, moderate in patients with cirrhosis, and dramatic in patients with HCC [8]. This indicates that the performance of GP73 might depend on the etiology of the underlying disease. This is important if one wants to differentiate between non-malignant disease and early HCC in; for instance, patients with chronic viral hepatitis. The authors also claimed tumor recurrence to be correlated with an elevated GP73 level in the blood [8]. Thus, besides being an interesting screening test, GP73 might also be useful as a surveillance test. The role of intrahepatic metastasis of the original tumor versus the development of de novo tumors could not be tested by Mao et al. The authors found no effect based on tumor size and tumor differentiation on the serum levels of GP73 [8].

The small amount of data available per paper on differences in etiology, tumor recurrence, and tumor development (numbers of tumors) precluded us from establishing the performance of GP73 in relation to these three parameters.

It would be interesting to examine whether combined measurements of GP73 and AFP further increase the sensitivity for detection of HCC. Although GP73 is a promising marker, more studies are warranted, especially because this protein is detected by Western blot analysis which hampers its reliability and availability in clinical use. Further studies are needed to analyze and validate early-stage HCC markers. Recently, Shang et al. [15] evaluated osteopontin as a marker of early-stage HCC. Although this study has some limitations, it is an important first step in the evaluation of new markers of early-stage HCC [15]. The next step should be large-scale validation to determine whether osteopontin is superior to GP73 and to analyze whether osteopontin in combination with GP73 complements screening tests.

In conclusion, GP73 is a valuable serum marker that is superior to AFP and can be useful in the diagnosis and screening of HCC. GP73 may improve the detection and treatment of one of the most common malignancies worldwide. More studies are needed to further elucidate the influence of the etiology of disease on the signal strength of GP73.

## Abbreviations

HCC: Hepatocellular carcinoma
AFP: Alpha-fetoprotein
GP73: Golgi protein 73
PIVKA II: Prothrombin induced by vitamin K absence II
sROC: Summary ROC
AUC: Area under the curve
IL-6: Interleukin-6
SCCA: Squamous cell carcinoma antigen
RU: Relative units
